# Metformin Downregulates the STAT Pathway and Reduces Bone Marrow Fibrosis in Primary Myelofibrosis Patients: Final Results of the Phase II FIBROMET Trial

**DOI:** 10.1002/hon.70163

**Published:** 2025-12-28

**Authors:** Paula de Melo Campos, Kátia Borgia Barbosa Pagnano, Fernanda Soares Niemann, Rubia Isler Mancuso, Fernanda Isabel Della Via, Ada Congrains, Juan Luiz Coelho‐Silva, Ângela Condotta Tinoco, Guilherme Rossi Assis‐Mendonça, Leandro Luiz Lopes de Freitas, Fabiola Traina, Sara T. Olalla Saad

**Affiliations:** ^1^ Hematology and Transfusion Medicine Center Universidade Estadual de Campinas (Unicamp) Sao Paulo Brazil; ^2^ Department of Medical Imaging Hematology and Oncology Ribeirão Preto Medical School University of São Paulo Ribeirão Preto Brazil; ^3^ Department of Pathology Universidade Estadual de Campinas (Unicamp) Sao Paulo Brazil

**Keywords:** FIBROMET, fibrosis, JAK‐STAT, metformin, myelofibrosis

## Abstract

Primary myelofibrosis (PMF) is a chronic myeloproliferative neoplasm characterized by the activation of the JAK‐STAT pathway. Previous evidence showed that metformin might be a possible therapeutic option for treating JAK2‐mediated myeloproliferative neoplasms. In vitro and in vivo studies demonstrated that metformin inhibits the JAK‐STAT pathway, induces apoptosis in JAK2^V617F^‐positive cell lines and reduces tumor burden and splenomegaly in Jak2^V617F^ knock‐in‐induced mice. The FIBROMET trial, an open label phase II study, evaluated metformin effects on 10 primary myelofibrosis patients over 2 years of treatment. Primary endpoint was bone marrow fibrosis reduction. Secondary endpoints were constitutional symptoms, blood counts, spleen size modulation and exploratory evaluation of protein and gene expression. Metformin treatment reduced bone marrow collagen deposits, downregulated the STAT pathway and reduced the p85 subunit of PI3K enzymatic complex, together with endothelial maintenance genes, in PMF patients. These results raise new evidence regarding metformin, a cheap and widely available drug, as a possible adjuvant for the treatment of PMF patients.

## 1 Introduction

Primary myelofibrosis (PMF) is a chronic myeloproliferative neoplasm characterized by the presence of clonal proliferation of myeloid cells in the bone marrow (BM) led by driver mutations, which include *JAK2*, *MPL* and *CALR*, that induce disease phenotype most notably by the activation of the JAK‐STAT pathway [1, 2]. Additional mutations in genes such as *ASXL1*, *SRSF2*, *U2AF1*, *IDH1*, *IDH2*, *EZH2*, *DNMT3A*, among others, are also reported and might influence disease progression [1]. After an early stage of prefibrotic primary myelofibrosis, the clonal proliferation results in fibrosis and leads to BM failure, resulting in a range of clinical manifestations, such as constitutional symptoms, anemia and hepatosplenomegaly [1, 2, 3]. The exact mechanisms of BM fibrosis are unknown, however, abnormal megakaryocytes may have a role in stimulating cytokine secretion, such as TGF‐β, PDGF and FGF from the granules of engulfed cells, resulting in fibrosis, neoangiogenesis, and osteosclerosis [2, 4, 5]. The JAK‐STAT pathway is the main signaling mechanism for a wide array of cytokines and growth factors that are dysregulated in PMF patients [6].

Metformin (MTF) is a medication from the biguanide class that mainly acts by decreasing hepatic glucose production through gluconeogenesis inhibition, resulting in potent antihyperglycemic effects. MTF mechanism of action involves a mild and transient inhibition of the mitochondrial respiratory chain complex I, resulting in the activation of AMPK, a cellular metabolic sensor [7]. Epidemiological evidence provided by large cohort studies have associated persistent elevated plasma glucose levels, insulin resistance and the resultant hyperinsulinemia with an increased risk of cancer [7], while MTF resulted in reduced risk of cancer and cancer‐related mortality [8, 9]. Evidence from in vitro studies suggest that MTF attenuates tumor growth by activating AMPK‐mTOR signaling and by p53 up‐regulation [10, 11]. Furthermore, MTF may reduce the Insulin/IGF‐1 levels, thereby inactivating its downstream PI3K/Akt/mTOR signaling pathways to inhibit tumor cell proliferation [10, 12]. Finally, an AMPK‐dependent inhibition of JAK/STAT signaling has also been decribed [13]. In cell lines harboring JAK2^V617F^ mutation, MTF induced apoptosis, reduced cell viability, inhibited JAK‐STAT pathway and enhanced ruxolitinib antileukemic effects [14, 15]. In Jak2^V617F^ knock‐in‐induced MPN mice, MTF reduced tumor burden and splenomegaly [15].

Given the promising effects of MTF observed in JAK2^V617F^ cell lines and mouse models, we hypothesized that MTF could be an effective treatment for PMF patients. We therefore undertook an open label phase II study to evaluate MTF effects on BM fibrosis, inflammation mediators and JAK‐STAT pathway activation in PMF patients (FIBROMET trial).

## 2 Methods

The phase II FIBROMET trial included patients with the diagnosis of PMF followed at the Hematology and Transfusion Medicine Center—Universidade Estadual de Campinas (Unicamp), Brazil, from August 2018 until February 2021. Inclusion criteria included: PMF diagnosis according to WHO criteria confirmed by at least two independent specialists, age ≥ 18 years‐old, glomerular filtration rate > 60 mL/min and adequate liver function (bilirubin < 1,5x and AST/ALT < 3x superior limit). Exclusion criteria included: biguanide hypersensitivity, diagnosed diabetes mellitus, chronic alcohol intake, untreated vitamin B12 deficiency, pregnancy or breastfeeding, ongoing HIV or hepatitis B/C infection, active autoimmune diseases, uncontrolled comorbidities, severe psychiatric disorders and anagrelide and/or ruxolitinib use in the 60 days that preceded screening. Primary endpoint was bone marrow fibrosis reduction. Secondary endpoints were constitutional symptoms, blood counts, evaluation of clonal mutations, inflammation mediators and protein and gene expression modulation.

Patients were started on 500 mg of slow‐release MTF per day and doses were increased by 500 mg weekly until reaching 2500 mg daily or the maximum tolerated dose. Treatment was maintained for 2 years or until the emergence of progressive disease, intolerable side effects or limiting comorbidities. Clinical and laboratory evaluation was performed at screening and after 3, 6, 12, 18 and 24 months on MTF treatment. Laboratory and clinical data were collected by analysis of electronic medical records. The extent of BM collagen deposits was semi‐quantitatively assessed by reticulin and Masson's trichrome staining, following recommendations of the European Consensus on grading of BM fibrosis [16] (grades 0, 1, 2, or 3). All the slides were independently evaluated by two hematopathologists, and discordant cases were submitted to joint review. The Second Harmonic Generation (SHG) signal was captured by a non‐descanned detector at 495–560 nm (green) and 560–620 nm (red). The images were obtained from hematoxylin‐eosin slides obtained from 20 μm thick tissue slices using an inverted LSM780 confocal/multiphoton microscope (INFABIC, Unicamp). The autofluorescence signal was subtracted from the SHG image. For this analysis, three fields were examined per slide at 40x magnification. Three‐dimensional collagen analysis was conducted using ImageJ/Fiji. Only collagen signals (F and B) were retained, and their integrated density was quantified in three areas of interest (AOI) for each image, measuring 150 × 150 nm pixels each. Fiber organization (coherency) and proximity (uniformity) were evaluated with the OrientationJ plugin using the same AOIs. STAT pathway (pSTAT3/pSTAT5) activation was assessed by phospho‐flow cytometry. Plasma cytokine expression was evaluated by Multiplex assay. Insulin signaling gene modulation was performed by PCR Array (PAHS‐030Z, Qiagen). Clone modulation was assessed by next generation sequencing using the Agilent Haloplex kit customized for 89 genes previously related with myeloid malignancies and analysis was performed according to ACMG Guidelines [17]. For somatic variant identification, tumor and buccal swabs DNA sequencing results were assessed to overlap variant profiles. When variant allele frequency in buccal DNA was < 45%, the variant was assigned as somatic for clonal evolution analysis.

This trial was approved by the Institutional Review Board from the School of Medical Sciences of Unicamp, Brazil (CAAE: 86624318.3.0000.5404) and registered at the Brazilian Registry of Clinical Trials (REBEC RBR‐52ty66). Written informed consent was obtained from all subjects. All procedures were performed in accordance with the Declaration of Helsinki.

## 3 Results

Eleven patients were recruited. One patient had an early drop out from the study after a femur fracture and did not begin MTF use. Ten patients were included: five women and five men, aged between 41 and 84 years (median 73 years old). The duration of MTF use varied from 3 to 24 months, with a median treatment persistence of 21 months. A single patient had an early MTF discontinuation after 3 months due to an unrelated cause (death by neurocysticercosis). MTF was well‐tolerated and adverse events were manageable, with the maximum tolerated dose ranging from 1500 to 2500 mg. Diarrhea was the most common adverse event observed (5 out of 10 patients): three patients had resolution of the symptoms after reducing the dose to 1500 mg per day and two of them had spontaneous remission of the symptoms without dose modification. No episodes of hypoglycemia were noted. Baseline characteristics and response to treatment are summarized in Table 1.

**TABLE 1:** Clinical and laboratory characteristics of primary myelofibrosis patients included in the FIBROMET trial throughout metformin treatment.

							Screening							Data at MTF discontinuation					
Patient ID	Age	Sex	Date of diagnosis	Time on MTF (months)	MTF MTD	MTF side effects	BM fibrosis	Hb	WBC	ANC	PLT	PB blasts	DIPSS	BM fibrosis	Hb	WBC	ANC	PLT	DIPSS
1	74	F	03/05/08	24	2500	No	0	13.9	2930	1530	399,000	0	INT‐1	0	13.2	5340	3630	791,000	INT‐1
2	67	M	02/27/18	3	2500	No	3	13.5	12,520	9980	136,000	0	INT‐1	3	11.4	9080	5175	169,000	INT‐2
3	84	M	05/27/15	6	2500	Diarrhea	3	7.1	9700	7490	619,000	1	High	3	7	8010	5990	520,000	INT‐2
4	77	F	07/22/15	24	2500	Diarrhea	1	13.8	3990	2350	218,000	0	INT‐1	0	12.9	3710	2540	576,000	INT‐1
5	44	M	03/28/07	12	1500	Diarrhea	2	13.1	3220	2040	256,000	0	Low	3	9.9	6770	5145	290,000	INT‐2
6	41	F	08/30/05	24	1500	Diarrhea	0	12.6	3460	1610	905,000	0	Low	0	12.5	2960	1390	778,000	Low
7	54	F	04/17/13	24	2500	No	0	13.4	4660	2860	143,000	0	Low	1	13.4	5910	4060	284,000	Low
8	73	M	10/22/13	18	2500	No	1	12.5	5540	3870	810,000	0	INT‐1	1	11.1	4610	3580	885,000	INT‐1
9	77	M	12/02/09	18	2500	No	2	11.4	4480	3225	585,000	1	INT‐2	3	10.6	11,370	6822	338,000	INT‐2
10	73	F	07/19/18	24	1500	Diarrhea	3	10.9	11,370	9300	94,000	0	INT‐1	2	10.5	7850	5950	63,000	INT‐1

*Note:* (1) Treatment interruption: (2) death by neurocysticercosis, (3) poor adhesion to treatment, (5) PMF progression; submitted to BM transplantation, (8) diagnosis of colon cancer, (9) pulmonary thromboembolism.

Abbreviations: ANC: absolute neutrophil count (/mm^3^), DIPSS: Dynamic International Prognostic Scoring System, INT‐1: Intermediate‐1, INT‐2: Intermediate‐2 Hb: Hemoglobin (g/dL), MTD: maximum tolerated dose (mg); NA: not applicable, out of treatment; NP: not performed; PLT: platelet counts (/mm^3^), Sex: F (female)/M (male); WBC: Leukocytes (/mm^3^).

**FIGURE 1:**
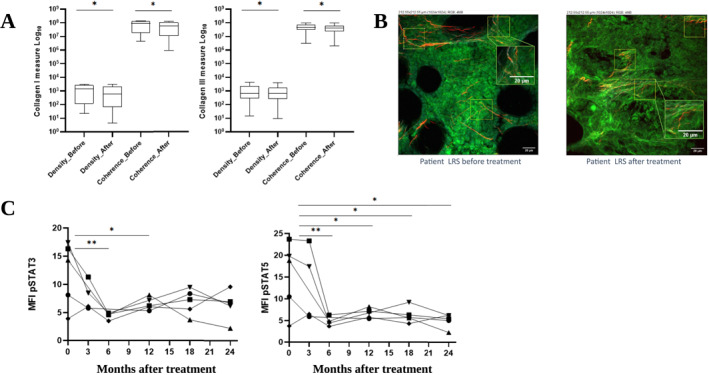
Metformin modifies collagen loading and structural organization in primary myelofibrosis (PMF) patients and reduces STAT3 and STAT5 phosphorylation. (A) Remodeling of bone marrow collagen matrix after 24 months of metformin treatment evaluated by second harmonic generation. A significant decrease in the density and coherence of the fibers for both collagen types I and III (F and B) was noted. (B) Bone marrow collagen (red channel) demonstrated by second harmonic generation microscopy in a PMF patient treated with metformin for 24 months. A reduction both in density (quantity) and organization of the collagen fibers is seen when comparing pre and post treatment samples. (C) STAT pathway downregulation after metformin treatment evaluated by phospho‐flow cytometry assay. Lines represent individual patients over treatment time. MTF reduced intracellular STAT3 phosphorylation after 6 and 12 months of treatment, and STAT5 phosphorylation after 6, 12, 18 and 24 months of treatment. *p ≤ 01; **p ≤ 05.

Analysis of the BM collagen matrix using SHG was performed at baseline and after 24 months in the five patients who completed the study, revealing significant remodeling following 24 months of metformin treatment (Figure 1A). For both collagen types I and III, a statistically significant decrease in density was observed (*p* = 0.0313), indicating a reduction in the total amount of fibers. In parallel, the coherence of the fibers, which reflects their organization and alignment, also presented a significant change for both collagen types (*p* = 0.0313); specifically, post‐treatment samples presented less organized fibers (Figure 1B). Collectively, these findings suggest that MTF treatment impacted BM fibrosis not only by reducing the collagen load but also by modifying its fibrillar structure. No changes in BM collagen deposits were observed during MTF treatment as assessed by Masson's trichrome staining across the different time points (samples were compared at time points 0 and 3 months, for one patient; 0, 3 and 6 months, for one patient; 0, 3, 6 and 12 months for one patient; 0, 3, 6, 12 and 18 months for two patients; and 0, 3, 6, 12, 18 and 24 months and for five patients). No significant differences were detected over time in splenomegaly, constitutional symptoms, or blood counts.

Metformin treatment led to the downregulation of the JAK‐STAT pathway by phospho‐flow cytometry assay. Five cases could not be evaluated due to unavailability of samples. In three cases, samples were not collected at all time points (early study discontinuation at 3, 6 and 12 months—deceased by neurocysticercosis, poor adhesion to treatment and BM transplantation, respectively) and, in two cases, the samples got degraded and could not be used. In the five cases that were fully assessed, STAT3 phosphorylation exhibited a gradual decline over the course of the study. Statistical analysis using two‐way repeated measures ANOVA revealed a significant temporal effect (F (5,18) = 3.601; *p* = 0.0197), which explained 43.63% of the total variance. Post‐hoc testing confirmed marked reductions in phosphorylation levels at 6 months (4.40 ± 0.24; *p* = 0.0188) and 12 months (6.31 ± 0.47; *p* = 0.0409), when compared to screening values (12.01 ± 2.58). No significant differences were observed at 3, 18, or 24 months. The patient factor and the interaction term (time × patient) were not significant, suggesting a consistent trend across subjects. Phosphorylation of STAT5 followed a similar pattern. Significant effects were observed for both time (F (5,18) = 6.090; *p* = 0.0018) and inter‐patient variability (F (4,18) = 3.258; *p* = 0.0355), accounting for 50.46% and 21.59% of the variance, respectively. Post‐hoc comparisons revealed significant decreases from baseline (15.32 ± 3.59) to nearly all subsequent time points: 6 months (4.67 ± 0.45; *p* = 0.0188), 12 months (6.25 ± 0.58; *p* = 0.0230), 18 months (6.24 ± 0.81; *p* = 0.0393), and 24 months (4.98 ± 0.72; *p* = 0.0227) (Figure 1C).

**FIGURE 2:**
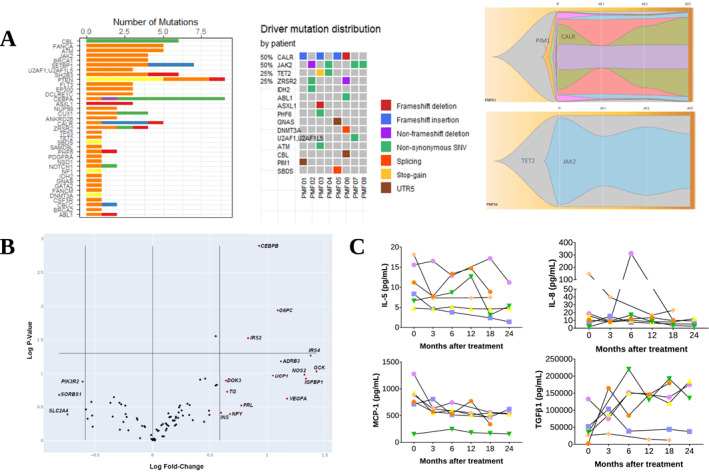
Molecular effects of metformin on primary myelofibrosis patients included on FIBROMET trial. (A) Mutation distribution and clone burden. Number of mutations and mutation distribution per patient are shown. We detected, overall, 66 non‐synonymous single‐nucleotide variants (SNVs), 64 mutations in 5′UTR regions, 35 insertions/deletions (17 frameshift indels) and 6 splicing mutations. Figures on the right exhibit clone burden throughout metformin treatment. Most clones were stable over time; however, we observed a modest reduction in clone burden in two of the patients 3 months after metformin treatment ‐ subtle bottlenecks are shown in these 2 patients, though not correlated with clinical parameters. (B) PCR Array gene expression modulation. Volcano plot depicting the extent (x‐axis) and significance (y‐axis) of differential gene expression for each target before (control group) and after 6 months of metformin (test group). Genes demonstrating ≥ 1.5‐fold in either direction, compared to control group, are highlighted in blue (downregulated) or red (upregulated) test group. (C) Cytokine expression in bone marrow plasma. IL‐5, IL‐8, MCP‐1 and TGF‐beta levels in the BM from patients at screening and after 3, 6, 12, 18, and 24 months after MTF treatment are shown. Each color represents a patient during the treatment. No significant difference on cytokine levels was observed.

As expected, driver mutations in *JAK2* (50%) and *CALR* (50%) were the most frequently found. No mutations in *MPL* were identified. All patients presented additional driver mutations, with the most common being *TET2* (25%) and *ZRSR2* (25%). A modest reduction in clone burden was observed in two of the patients after 3 months of MTF treatment, however, most clones were stable over time. Mutation distribution and clonal dynamics are shown in Figure 2A.

PCR Array gene expression modulation was observed following MTF treatment: three genes were downregulated and 15 were upregulated after 6 months on MTF (Figure 2B). Among differentially expressed genes in the insulin‐related pathway, two insulin receptor substrates (*IRS2* and *IRS4*), the insulin gene (*INS*), and *VEGFA* were all upregulated after 6 months of treatment. Of note, downregulated genes were the glucose transporter (*SLC2A4*), the p85 subunit of PI3K enzymatic complex (*PIK3R2*), and the C‐Cbl associated protein related to insulin cellular stimulation (*SORBS1*).

The analysis of plasma cytokines expression showed a slight reduction in MCP‐1, IL‐5 and IL‐8 levels, and an increase trend in TGF‐beta levels in 3 of 6 patients after 24 months of treatment, though not statistically significant (Figure 2C). No differences were observed in the levels of CXCL4, sIL‐2RA, IL‐15, IL‐18, VEGF, MIP‐1alpha, MIG, IL‐1RA, IL‐6, TNF‐alpha, and IP‐10 (data not shown).

## 4 Discussion

Metformin is a cheap, widely available medication, that is regularly prescribed for patients with diabetes mellitus. MTF downregulates the PI3K/Akt/mTOR signaling pathways and inhibits tumor cell proliferation in a wide range of tumors, some of them of hematologic origin [10, 12, 18, 19]. Recently, Hosseini and colleagues demonstrated an elevated mitochondrial metabolism as a driver of clonal hematopoiesis, which can be counteracted by MTF in *Dnmt3a* ^‐^mutated cells in mouse models, reducing the competitive advantage of clonal cells [20]. Previous data showed evidence that MTF might also be a possible therapeutic option for treating JAK2‐mediated myeloproliferative neoplasms. Kawashima et al. [14] described that MTF inhibits JAK‐STAT pathway, induces apoptosis and enhances ruxolitinib suppressive action on cell growth in JAK2^V617F^‐positive HEL and SET2 cell lines. Phenformin, another drug from the biguanide class, reduces cell viability and increases apoptosis in SET2 JAK2^V617F ^cells [21]. Machado‐Neto et al. described that, in Jak2^V617F^ knock‐in‐induced MPN mice, MTF reduces tumor burden and splenomegaly [15]. A population study performed in Denmark with 3816 patients with MPN and 19,080 age and sex‐matched controls found a decrease in the incidence of MPN in the subgroup of long‐term MTF users (≥ 5 years), suggesting that MTF might have protective effects [22].

The phase II FIBROMET trial evaluated MTF effects on primary myelofibrosis patients, in which JAK‐STAT pathway is described to be upregulated [1]. MTF dose was escalated weekly until reaching 2500 mg per day or the maximum tolerated dose, since there was no dose‐finding study. MTF was generally well tolerated without unmanageable adverse effects; however, three of 10 patients were unable to tolerate the planned dose escalation. Flow cytometry data indicated that MTF can downregulate the STAT pathway in PMF patients. Also, we found that MTF reduced BM fibrosis collagen load and modified its fibrillar structure when evaluated by second harmonic generation, which is a more sensitive method than the Masson's trichrome. Furthermore, PCR Array gene expression revealed a downregulation of the gene of the p85 subunit of PI3K enzymatic complex, together with endothelial maintenance genes.

These results are in line with the previous in vitro and in vivo studies found in literature and raise new insights regarding MTF, a cheap and widely available drug, as a possible adjuvant for the treatment of PMF patients. Although we did not observe clinical improvements in patients regarding blood counts and clinical presentation, we think that the small casuistic and the short follow up in some cases might limit definitive conclusions. Although metformin might be a promising adjuvant treatment for PMF patients, the results should be interpreted cautiously until confirmed in a further prospective larger cohort.

## Author Contributions

P.de.M.C., K.B.B.P., F.T. and S.T.O.S. contributed to the study conception and design, clinical evaluation and results' analysis. R.I.M. and F.S.N. contributed to cytokine and fibrosis experiments and analysis. F.I.D.V. and F.S.N. contributed to phospho‐flow cytometry experiments and analysis. A.C.T. contributed to next generation sequencing and clonal evolution analysis. J.L.C.S. contributed to P.C.R. Array gene expression analysis. A.C.T. contributed to slides preparation and immunohistochemistry for BM biopsy evaluation. G.R.A.M. and L.L.L.de.F. performed bone marrow evaluation and fibrosis classification. All authors contributed to manuscript writing and editing. All authors read and approved the final manuscript.

## Funding

This study was supported by Conselho Nacional de Desenvolvimento Científico e Tecnológico (CNPq), Brasil (Grant 303405/2018‐0), São Paulo Research Foundation (FAPESP), Brasil (Grant 2017/21801‐2), and Instituto Nacional de Ciência e Tecnologia do Sangue (INCTS), Brasil (Grant 405918/2022‐4).

## Ethics Statement

This trial was approved by the Institutional Review Board from the School of Medical Sciences of the University of Campinas, Brazil (CAAE: 86624318.3.0000.5404) and registered at the Brazilian Registry of Clinical Trials (REBEC RBR‐52ty66). Written informed consent was obtained from all subjects. All procedures were performed in accordance with the Declaration of Helsinki.

## Conflicts of Interest

The authors declare no conflicts of interest.

## Data Availability

The data that support the findings of this study are available on request from the corresponding author. The data are not publicly available due to privacy or ethical restrictions.
